# The Children's Hospital, Birmingham

**Published:** 1890-04-05

**Authors:** 


### The Hospital Workshop.

(BY OUR SPECIAL COMMISSIONER.)

### No. XXIV.—THE CHILDREN'S HOSPITAL, BIRMINGHAM.

Nearly all Birmingham houses are built of brick, and the hospitals are no exception to the prevailing fashion. In the hands of a skilful architect the material is capable of an infinite variety of arrangement, and, when mellowed by time, presents a rich colouring that is rarely met with in stone. The Children's Hospital shows some good brickwork, the more ornamental being in the detached wards that lie at the back, and cannot be seen from the outside. Its main block, of three storeys, faces Broad Street, and was originally a lying-in charity, converted into an out-door attendance some twenty years back. This block contains half-a-dozen wards, together with committee-rooms and residential quarters for the two house surgeons and other officials. The whole placeaffords accommodation for about eighty patients, who are tended by nineteen nurses and a lady superintendent.

Under the courteous guidance of the senior house surgeon, Mr. H. L. Ewens, your Commissioner lately visited this institution, and is enabled to lay the following sketch before readers of The Hospital. The wards in the main block are bright and cheerful, and have exactly that air of comfort which sick children require. The walls, of pearl-grey distemper over a green dado, are hung with pictures, mirrors, and brackets, while around are flowers, plants, toys, goldfish, and birds. The windows, fitted with Venetian blinds, lie on opposite sides of the ward, and, being carried down almost to the floor, admit a liberal allowance of light. Near the entrance of each ward is painted up a notice of the contained cubic space. The "male surgical," for instance, has 3,074 cubic feet, an allowance of 734 cubic feet per bed. In the centre of the room mentioned is a large and handsome table of light oak, the gift of a late physician, Dr. Heslop. Most of the furniture in this ward, including the heavy brass railing fenders, and much in other parts of the institution, came from the same source. The cots are modern and convenient. Their sides either slide down a rod or fall open with a hinge motion. They are enamelled in light colours, and fitted with straw palliasse and hair mattress. This description of the furniture applies generally to the rest of the wards, as well as the fact that each has its own bath-room and lavatory.

Above the entrance hall on the first floor is a play-room cut off from the corridor by massive brass railings. A passing glance revealed a rocking-horse of great size and beauty, a weighing chair, and a big model of a life-boat, along with other objects of entrancing interest too numerous to mention.

Stretching away from the main block at a right angle, and lying deeply amongst the surrounding buildings, is a large brick-paved space containing the detached wards. The latter, four in number, from their external appearance might be a series of private chapels. Each has a lobby, containing, amongst other things, a fire extinguishing apparatus and a speaking tube to the main block. The interior of these wards is of an extremely striking and effective character. The floors are of polished oak, and the ceilings lofty. The walls are lined with square white tiles, having an upper and a lower dado of octagonal-shaped white tiles, each marked off by a narrow coloured border. The cots have turkey-red coverlets, and further brightness is afforded by the fine show of arum lilies and other blooms displayed on the centre table. Many of these flowers are sent by residents in the neighbourhood, and others, according to a graceful custom, are the result of special flower services held in the various churches. Heating is effected by means of an open fire-place, and a system of hot-water pipes at each end of the ward. Ventilation appears to be exceptionally well provided for, and is carried on by windows, by valves in the walls, by a gas-burner exhaust-shaft in the ceiling, and by outlet gratings at various points. Fire-buckets stand in each ward, and supplement a small hand-engine and "grenades." A hose used for washing the courts is also available, but hardly takes the place of a regularly-appointed fire-hose connected with the mains. Of the detached buildings, one belongs to the nurses. Another—holding four beds—is for cases that develop infection after admission; while a third, of small size, is reserved for diphtheria. The larger wards have an average of fourteen beds apiece. In one of them, a lad of thirteen lay still and motionless. Originally of a tubercular tendency, he has suffered from rheumatic fever, followed by pyæmia, and many abscesses have been opened from time to time in different parts of his body. The poor boy is almost literally skin and bone. His face—to use no mere figure of speech—is white as the pillow on which it lies, but the drawn mouth relaxes into a smile as the house surgeon cheers him with a few kind words. His temperature shows the typical irregularity of hectic fever. Generally higher at night, it has ranged up and down for months from subnormal to 102 deg. F., and even on one occasion to 103·5 deg. F.

Broadly speaking, none but acute cases are admitted, but this rule is elastic. There is a good deal of typhoid, of tuberculosis, of empyema, and a number of accidents. Heart disease is common, and, curiously enough, much more frequent among the girls. Many of the maladies are the result of improper feeding, and some startling instances were mentioned by the House Surgeon. One child of a year and eight months old weighed no more than seven and a half pounds on admission. The poor little creature improved considerably under treatment, but was taken away by the mother, contrary to advice. On being brought to the hospital a second time, the child died within a week after admission. As a warning to the parents, a death certificate was refused, and the case referred to the coroner, who severely censured their conduct. One tiny child of fifteen months, pointed out as a case of infantile atrophy, in all probability due to wrong feeding, weighed some ten or twelve pounds. It should be added, however, that the mother was reported to be dying from acute phthisis. There seems to be a prevalent idea in Birmingham as to the virtues of strong tea for infants no less than for grown-up people. The simple rule most commonly followed in rearing children, even while yet in the cradle, is to let them have "whatever is going," so that considering the way patients have been brought up, and the fact that they come chiefly from the Black Country, results are wonderfully good. The detached wards at the back are actually in the shadow of a huge hardware factory, which is often at work by night as well as by day. In London one often hears the remark that it is almost impossible to get a house away from a railway line. In the Midland capital it is just as difficult to be out of sight of a factory.

During the year 1889 six hundred and fifty-four patients were treated in the wards. These figures, however, give but a small index as to the total amount of work done by the charity. In the out-patient department at Steelhouse Lane, about a mile away from the hospital, upwards of twelve thousand cases came under treatment during the same year.

